# Control of Particle Properties in Thermally-Induced Precipitation of Polyetherimide

**DOI:** 10.3390/polym15081944

**Published:** 2023-04-19

**Authors:** Laura Unger, Sybille Fischer, Jens P. W. Sesseg, Andreas Pfister, Jochen Schmidt, Andreas Bück

**Affiliations:** 1 Institute of Particle Technology, Friedrich-Alexander-Universität Erlangen-Nürnberg, Cauerstraße 4, 91058 Erlangen, Germany; 2 EOS GmbH Electro Optical Systems, Robert-Stirling-Ring 1, 82152 Krailling, Germany

**Keywords:** high-performance thermoplast, liquid–liquid phase separation, microparticles, precipitation, process control

## Abstract

The feasibility of thermally-induced phase separation and crystallization for the production of semi-crystalline polyetherimide (PEI) microparticles from an amorphous feedstock has been reported recently. Here, we investigate process parameter dependencies for designing and control of particle properties. A stirred autoclave was used to extend the process controllability, as the applied process parameters, e.g., stirring speed and cooling rate, were adjusted. By increasing the stirring speed, the particle size distribution was shifted to larger values (correlation factor ρ = 0.77). Although, the enhanced droplet breakup, induced by the higher stirring speed, led to the formation of smaller particles (ρ = −0.68), broadening the particle size distribution. The cooling rate showed a significant influence on the melting temperature, reducing it with a correlation factor of ρ = −0.77, as confirmed by differential scanning calorimetry. Lower cooling rates led to larger crystalline structures and enhanced the degree of crystallinity. The polymer concentration mainly affected the resulting enthalpy of fusion, as an increased polymer fraction enhanced the latter (correlation factor ρ = 0.96). In addition, the circularity of the particles was positively correlated to the polymer fraction (ρ = 0.88). The structure assessed via X-ray diffraction, was not affected.

## 1. Introduction

As the significance of additive manufacturing (AM) processes is increasing and the fields of application are growing, there is a demand for a wider range of suitable feedstock materials. Depending on the AM technique, different requirements have to be met when developing new materials. For example, for laser-based Powder Bed Fusion of Polymers (PBF-LB/P), one of the main challenges is the homogeneous deposition of the powder material on the building platform, typically performed by a blade or a roller coater. After the deposition, the defect-free layer is selectively melted via laser irradiation in the areas corresponding to the cross-section of the part. Afterwards, a fresh powder layer is applied on top and the procedure is repeated until the part is completely produced [1,2,3].

To address the challenge of powder deposition, polymer powders have to meet certain specifications concerning particle and bulk solid properties, referred to as extrinsic properties in the context of PBF-AM. These include the particle shape and size distribution as well as the bulk density and flowability [1,2,3,4,5]. In PBF-LB/P, the preferred powders are potato-shaped or spherical, and are characterized by narrow particle size distributions and an average particle size in the range of 50 to 100 µm [6,7,8,9].

For commercially available thermoplastic PBF materials, the most common feedstocks are polyamides, such as PA6, PA11, or PA12, with PA12 having the largest market share of more than 90% [10]. Similar to subtractive manufacturing approaches, the properties of the final part, such as mechanical strength and dimensional accuracy of the additively manufactured components, are mainly dependent on the properties of the feedstock material, assuming optimized process parameters. However, certain industries, such as medical applications, aviation, and aerospace, demand high standards regarding the mechanical, chemical and temperature resistance of their products. Therefore, it is vital to develop high-performance polymeric feedstock suitable to be processed in laser-based powder bed fusion, especially regarding the unique intrinsic flame retardancy of several high-performance polymers.

So far, the most common high-performance thermoplasts processed in PBF-LB/P are polyaryletherketones (PAEK), as many of them initially have a semi-crystalline structure. The prevalent manufacturing route for the production of particles is comminution, resulting in a large variety of (mainly) irregularly shaped particles. However, the processability is restricted unless post-processing steps are conducted in order to enhance the particle shape [10,11,12,13].

Among the production routes for polymeric microparticulate feedstocks for PBF-LB/P, comminution [14], liquid–liquid phase separation (LLPS) and melt emulsification [2] are the most common. Cryogenic comminution is the method applicable to the majority of polymers, allowing for a great variety of polymer powders. However, as already mentioned above, the resulting particles are characterized by an irregular shape. Melt emulsification and LLPS have the advantage of being able to generate close-to-spherical shapes with typically narrow size distributions, however, the identification of proper polymer–solvent systems suitable for these approaches is challenging [2].

LLPS and precipitation have been proven to be viable approaches to tailor both phase morphology and particle shape. For example, Kloos et al. demonstrated that an initially amorphous polycarbonate (PC) feed stock can be used to produce semi-crystalline PC microparticles of largely spherical shape and narrow size distribution [15]. Furthermore, the approach could be applied successfully for the production of polyoxymethylene (POM), polybutylene terephthalate (PBT), poly(L-lactide) (PLLA) and polyether ether ketone (PEEK) PBF-LB/P feed stocks [16,17,18,19]. Zhu et al. applied droplet evaporation-assisted thermally-induced phase separation for the production of PEI nanoparticles, using dimethyl sulfoxide solution (DMSO) as the liquid phase [20].

More recently, we were able to demonstrate that granule feedstock of amorphous polyetherimide (PEI), a high-performance thermoplast, can be transformed to semi-crystalline microparticles by thermal precipitation from acetophenone and dimethyl phthalate [21]. The aforementioned solvents were shown to impact the obtained thermal and structural product properties: a higher induced melting temperature and crystallinity were observed for the product precipitated from dimethyl phthalate. Particles precipitated from acetophenone showed a somewhat superior shape and particle size distribution with respect to the desired application. The experimental setup used in [21] was based on small-scale autoclaves showing several restrictions regarding the variation of important process parameters for thermal precipitation, such as a well-defined stirring or the variation of the cooling rate. Consequently, the bulk solid properties of the powders obtained, in particular the particle morphology, may not yet be optimal with respect to, e.g., shape, surface texture or particle size distribution. Within this study, the thermal precipitation of PEI in a stirred autoclave with a total volume of 3 L that allows for the precise variation of the most relevant process parameters, namely cooling rate and stirring speed during LLPS and precipitation, was investigated. The impact of the process parameters on the resulting particle properties is discussed and evaluated using a multivariate correlation analysis.

## 2. Materials and Methods

### 2.1. Production Approach for Polymeric Microparticles: Liquid–Liquid Phase Separation and Precipitation

**Figure 1:**
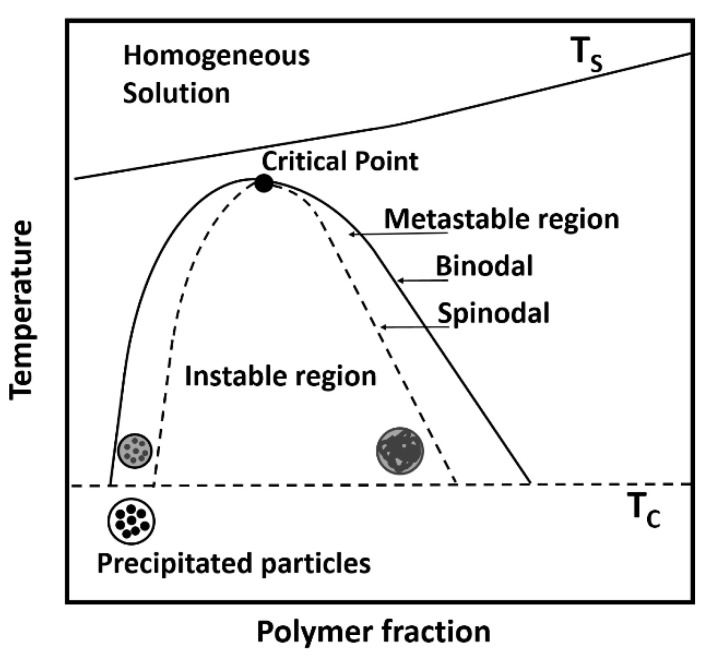
Simplified binary phase diagram for the polymer–solvent system (adapted from [28,29,30]).

The extrinsic properties of particles produced by LLPS and precipitation are beneficial for processability in the PBF-LB/P approach. Therefore, mainly close-to-spherical or potato-shaped particle shapes are produced, within a narrow particle size distribution. The crucial step in LLPS and precipitation is solvent selection, as a so-called moderate solvent is required. A simplified binary phase diagram for the system polymer-solvent is given in Figure 1, showing the principle of this process. A moderate solvent is a non-solvent under room temperature and a solvent at elevated temperatures. To ensure LLPS and precipitation, a homogeneous solution has to be prepared, which is achieved by heating the system above the polymer dissolution temperature. The latter is strongly dependent on the system composition, i.e., the polymer and solvent used and the respective concentrations [22,23].

Afterwards, the homogenous solution is cooled down slowly, and the metastable region is entered. After passing the binodal line, LLPS occurs, where the formation of a liquid-liquid dispersed system sets in, consisting of polymer-rich droplets in a polymer-lean continuous phase and, respectively, vice versa, depending on the selected polymer concentration. Membranes are obtained using high polymer concentrations, hence, polymer-lean droplets in a polymer-rich continuous phase are formed. The formation of polymer particles is triggered by the polymer-rich droplets in a polymer-lean continuous phase (systems with low polymer concentration). Upon further cooling in the metastable region, droplet coalescence or Ostwald ripening may occur. By passing the crystallization temperature T_C_, the particulate structures or membranes begin to precipitate as a result of the supersaturation of the polymer in the droplets or the continuous phase [24].

Considering the resulting particle size, a dependence of the former droplet size is well known in the literature [25,26]. Moreover, in dilute systems, the particle size grows with increasing polymer concentration [26,27].

### 2.2. Materials

**Figure 2:**
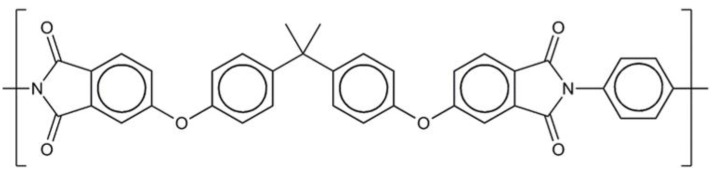
Structural formula of polyetherimide.

Polyetherimide feed material Ultem CRS5011 (Sabic, Riyadh, Saudi Arabia) provided as granules (height: 3 mm; diameter: 1 mm) was used. The structural formula of the repetition unit of the polyetherimide is given in Figure 2. The previously identified [21] acetophenone (ρ = 1.03 g cm^−1^, 99%, VWR, Atlanta, GA, USA) was used as the moderate solvent. For the post-processing steps, i.e., filtration and washing, denatured ethanol (96%, VWR, United States) was used.

### 2.3. Experimental Setup

For the parameter study, a stirred autoclave (Type 3E (Büchi AG, Flawil, Switzerland), 3 L, 200 bar) was used. A total amount of 2 L was used for the experiments. Stirring was carried out using an anchor-shaped stirrer (length: 190 mm; diameter: 90 mm), and the rotation speed for the experiments was varied between 350 and 500 min^−1^. A PID temperature control system monitored the heating and cooling periods, during which heating was carried out by an electric resistance heater and cooling was provided by a coolant circuit. The cooling rates used in the experiments varied between 1.5 and 3 Kmin^−1^.

The post-processing steps applied were identical. At a temperature of 60 °C, the reactor was opened, the obtained suspensions were washed using ethanol, and the precipitated solid was collected by filtration using a Büchner funnel (Grade 1 filter, Whatman, Maidstone, UK). The resulting particles were dried at 120 °C in ambient atmosphere in an oven UFP800DW (Memmert GmbH, Büchenbach, Germany). For the following analysis, sieving was performed using a mesh with a mesh size of 200 µm.

### 2.4. Experimental Plan

The experimental plan is given in Table 1 and Table 2. For the stirred autoclave a two-level factorial design of experiments (Table 1) was investigated.

As the stirred autoclave has an external cooling unit, defined cooling rates can be applied during the production of microparticles. Here, cooling rates of 1.5 and 3 K min^−1^ were considered, as those have been promising in previous studies [15,16,18]. Moreover, the installed anchor stirrer allows a precise control of the stirring speed, provided that the polymer–solvent mixture consists of a diluted system.

Based on the solvent screening study [21], polymer concentrations of 10 and 15 wt.% were chosen because the obtained particles showed preferable properties. The complete two-level factorial plan is shown in Table 2, giving the total number of experiments investigated.

### 2.5. Characterization Methods

#### 2.5.1. Scanning Electron Microscopy (SEM)

For analysis of particle shape and morphology, a SEM Ultra55 (Carl Zeiss AG, Oberkochen, Germany) was used, operating with a secondary electron detector for imaging at an acceleration voltage of 1.0 kV. The sample images were recorded at appropriate magnifications.

#### 2.5.2. X-ray Diffraction (XRD)

A powder diffractometer (Empyrean (Malvern Panalytical, Malvern, UK)) in Bragg–Brentano setup, equipped with a GaliPIX^3D^ detector was used for the structural analysis. The X-ray patterns were recorded within a 2 theta range of 3° to 60°, with a step width of 0.014°/step using Cu Kα radiation (λ = 0.15405 nm).

#### 2.5.3. Laser Diffraction Particle Sizing

Particle sizes and respective distributions were analyzed using a laser diffraction device (Mastersizer 2000 (Malvern Panalytical, Malvern, UK)) equipped with a Hydro 2000S wet dispersing unit. After preparing a suspension of powder, water, and a small amount of sodium dodecyl sulphate (SDS, 98% (Merck, Darmstadt, Germany)), the measurement was performed under stirring and ultrasonication. SDS was added to assure dispersion stability.

#### 2.5.4. Differential Scanning Calorimetry

A DSC Polyma 214 (Netzsch, Selb, Germany) was used for thermal characterization. For this, 10 mg of powder were placed in a standard aluminum pan. The sample was exposed to the previously defined temperature program, with heating and cooling rates of 20 K min^−1^ in a temperature range of 0 °C to 360 °C. The temperature program was repeated a second time, to ensure that the initially amorphous structure of the polyetherimide was observable.

#### 2.5.5. Particle Shape Analysis

Shape analysis was performed by static image analysis within an optical light microscope Axio Imager M1m (Carl Zeiss AG, Oberkochen, Germany). At the beginning of the measurement, particles were dispersed onto a transparent microscope slide to create a monolayer. Recording was performed in transmitted light mode using the Zeiss ZEN Core v3.2 software.

Afterwards, the extended particle analyzer of the Biovoxxel toolbox was implemented in Fiji software to obtain the shape factors of circularity and roundness, see Equations (1) and (2). A total of 15,000 particles were analyzed for each sample, whereby the particle size limit for the detection was set to the maximum value obtained from laser diffraction particle sizing (x_99,3_).

(1) $$c=\frac{2\sqrt{\pi A}}{P}$$

(2) $$r=\frac{4\cdot A}{\pi \cdot x_{max}^{2}}$$

The shape factors are in analogy with an ideal circle, as the observable maxima have a value of 1. Thus, the circularity gives a comparison of the particle aspect ratio and the roughness of the surface. Roundness, instead, solely depends on the particle aspect ratio. However, assuming a constant projected area, an increasing projected perimeter yields the formation of a star-shaped particle, decreasing the circularity. Roundness is decreased when the particle shape is evolving towards an ellipse, increasing the major axis.

#### 2.5.6. Powder Flowability

To characterize the powder flow, the ring shear cell tester RST-XS.s (Dr. Dietmar Schulze Schüttgutmesstechnik, Wolfenbüttel, Germany) was used. The applied consolidation stresses were 1200, 2600, and 4600 Pa. The ratio of consolidation stress σ_1_ to unconfined yield strength σ_c_ is defined as the flow function ff_c_, according to Jenike [31,32].

### 2.6. Multivariate Correlation

The multivariate correlation method was used to investigate the correlations between the three main components (polymer fraction, stirring speed, and cooling rate) and the resulting particle properties.

A Matlab code was used to evaluate the correlation of the normalized data sets by returning a matrix of the pairwise linear correlation coefficient between each pair of columns of the input matrices *X* and *Y*. In the following, the Pearson’s Linear Correlation Coefficient is used, returning a *k*_1_-by-*k*_2_ matrix, where *k*_1_ and *k*_2_ are the number of columns in *X* and *Y*, respectively. *ρ*(*a*,*b*) is the pairwise linear correlation coefficient between column *a* in *X* and column *b* in *Y*.

(3) $$\rho \left(a,b\right)=\frac{\sum_{i=1}^{n}\left(X_{a,i}-{\bar{X}}_{a}\right)\left(Y_{b,i}-{\bar{Y}}_{b}\right)}{{\left\{\sum_{i=1}^{n}{\left(X_{a,i}-{\bar{X}}_{a}\right)}^{2}\sum_{j=1}^{n}{\left(Y_{b,j}-{\bar{Y}}_{b}\right)}^{2}\right\}}^{1/2}}$$

where *n* is the length of each column, *X_a_* and *Y_b_* are the respective columns in the *X*–*Y* matrix, and ${\bar{X}}_{a}$ and ${\bar{Y}}_{b}$ are the respective means. The range of the correlation coefficient is −1 to +1, where a value of −1 indicates a perfect negative and a value of +1 indicates a perfect positive correlation. For *ρ*(*a*,*b*) = 0, no correlation between the variables is indicated.

## 3. Results and Discussion

### 3.1. Process Efficiency and Yield

The yield of the product is displayed in Table 3. The yield is defined as the powder mass remaining after post-processing is completed, e.g., filtering, washing, drying, and sieving. It thus represents the usable amount of polymer powder for further analysis, as given in Equation (4).

(4) $$\mathrm{Yield}=\frac{Total\;output\;mass}{Input\;mass}$$

For each sample, the entire product could be used for analysis without loss after post-processing. Considering the powder samples consisting of 10 wt.% polymer (No. 1–4) produced in the stirred autoclave, the minimal yield was 85.3%, whereas the maximum feasible is 93.6%, that is, the higher the stirring speed, the lower the yield. Additionally, the applied cooling rate showed a modest impact on the yield.

**Figure 3:**
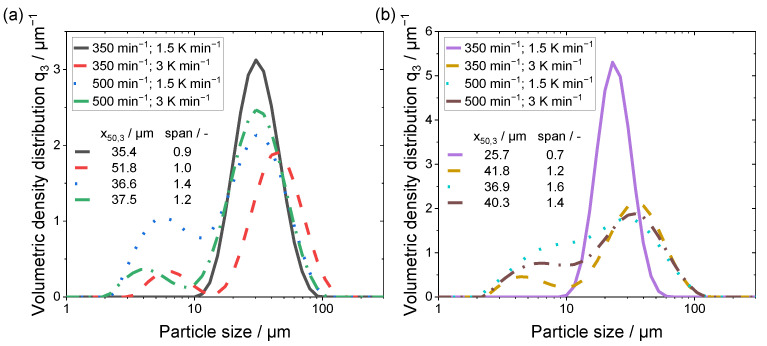
Volumetric density particle size distributions of (**a**) 10 wt.% and (**b**) 15 wt.% polymer fractions. Mean particle size x_50,3_ and span are given in the inset.

For the powders precipitated with a polymer concentration of 15 wt.% (No. 5–8), the yield was higher compared to the 10 wt.% samples, with a minimum of 91.9%. A yield of 100.62% can be observed for the 15 wt.% powder samples precipitated with a stirring speed of 350 min^−1^ at a cooling rate of 1.5 K min^−1^. The authors assume that either the superior high amount of median fractioned particles (resulting from the monomodal particle size distribution shown in Figure 3) or an incorporation of remaining solvent within the particles lead to the minimal surplus. As there is no discernible solvent peak in the curve of the respective DSC measurement, the amount of solvent has to be extremely low and should not interfere during processing.

### 3.2. Particle Size Analysis

The volumetric density particle size distributions are given in Figure 3a,b, separated with respect to the polymer fraction.

A noticeable divergence in the shape of the curves is displayed for both samples in Figure 3a,b, with the process parameters of 350 min^−1^ in combination with a cooling rate of 1.5 K min^−1^. The span exhibited has the smallest values (<1), however the mean particle size x_50,3_ is comparable to the other powder samples. Additionally, only this parameter combination yielded a monomodal distribution. Therefore, this process parameter combination shows the most promising particle size distribution, as the lower stirring speed induced less shear force during the droplet formation and growth processes leading to a monomodal size distribution with fewer fine particles.

The particle size distributions of the remaining powder samples exhibiting bimodal distributions are also shown. Thus, by an increased cooling rate, larger maximum particle sizes and a distinct amount of fine fraction are created. Furthermore, the combination of a higher stirring speed with the reduced cooling rate leads to a significant increase in the fine fraction and span of the distribution. The polymer concentration induces a shift towards larger particle sizes, revealed by the growing span.

By comparing the resulting mean particle sizes obtained in this work with the ones reports by Wang et al. [19], similarities can be found. The process parameters that Wang et al. used resemble the parameters used in the current manuscript. The rotation speed of the stirring unit was 600 min^−1^ and the cooling rate was 1 K min^−1^. The mean particles sizes for the precipitated PEEK microparticles showed a size of 56 µm, whereas the microparticles precipitated from PEI have mean particle sizes within a size range of 25.7 to 51.8 µm, depending on the process parameter applied.

### 3.3. Shape Analysis via SEM and Light Microscopy

**Figure 4:**
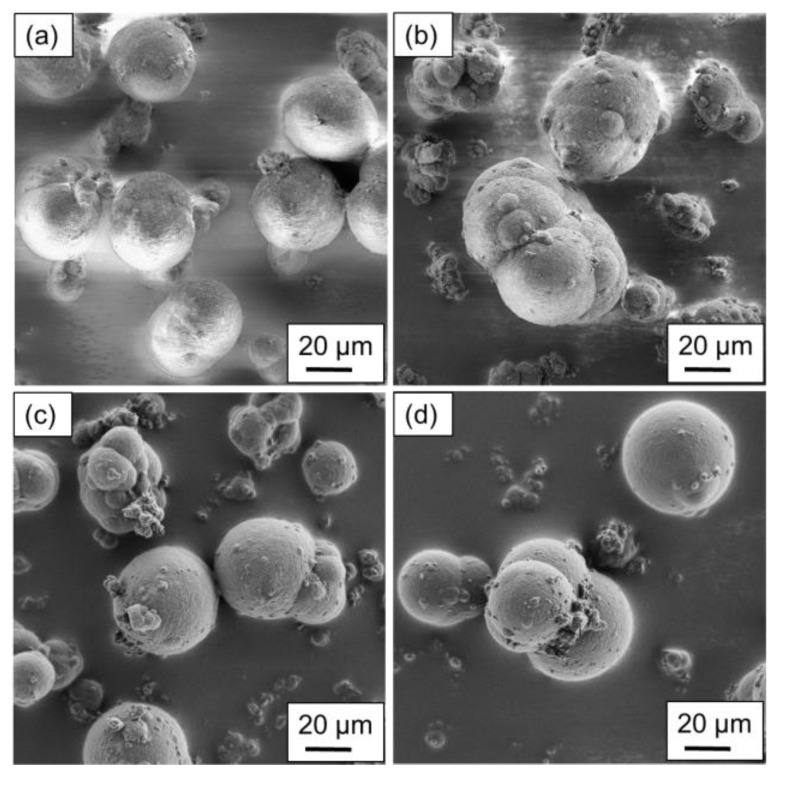
SEM images of the precipitated particles using a polymer concentration of 10 wt.% at process parameters of (**a**) 350 min^−1^ and 1.5 K min^−1^, (**b**) 350 min^−1^ and 3 K min^−1^, (**c**) 500 min^−1^ and 1.5 K min^−1^ and (**d**) 500 min^−1^ and 3 K min^−1^. The magnification used was uniform.

**Figure 5:**
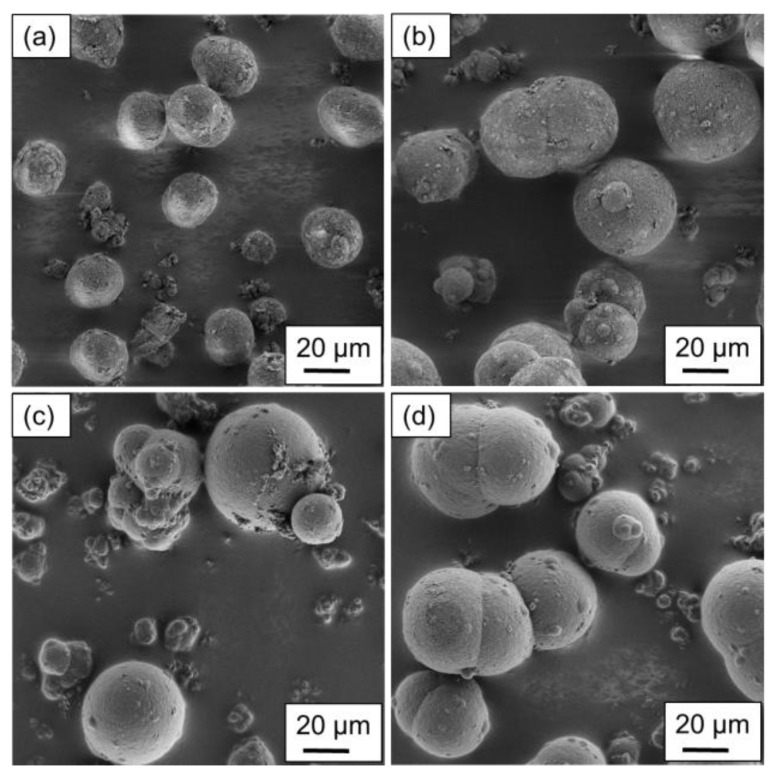
SEM images of the precipitated particles using a polymer concentration of 15 wt.% at process parameters of (**a**) 350 min^−1^ and 1.5 K min^−1^, (**b**) 350 min^−1^ and 3 K min^−1^, (**c**) 500 min^−1^ and 1.5 K min^−1^ and (**d**) 500 min^−1^ and 3 K min^−1^. The magnification used was uniform.

The particles produced using the stirred autoclave are shown in Figure 4 and Figure 5, where the variation in the process parameters is included in the caption. Figure 4a–d displays the particular structures resulting from a polymer concentration of 10 wt.%.

Regarding the shape analysis of single particles, potato- to spherical-shaped structures are found, depending on the parameter combination applied. In Figure 4a, a uniform particle distribution regarding the structure and size can be observed, aligning with the particle size measurements of the above section. With a higher cooling rate, a modest amount of particles smaller than 15 µm is distinguishable. The increased cooling rate limits the growth mechanism as the crystallization occurs prematurely. Furthermore, aggregated particles are not sufficiently coalesced, resulting in the formation of larger structures.

For Figure 4c,d the higher stirring speed was applied during the production, resulting in the formation of widely distributed structures and sizes. For the lower cooling rate (Figure 4c) the particles can have spherical as well as grape-shaped structures, as the stronger shear forces lead to an increased droplet breakup. However, the formation of spherical structures dominates, whereas grape-shaped structures can mainly be observed in smaller particles. By increasing the cooling rate, the nucleation time is decreased, whereas growth is increased, yielding in the formation of more spherical structures. However, the surface of the larger particles is partially covered with extremely small ones, as they aggregated to the larger particles after nucleation.

For the powder samples precipitated with a polymer concentration of 15 wt.%, similarities to the particles presented in Figure 4 can be identified. The most preferable shape is observed for the parameter combination used in Figure 5a, as a uniform distribution in size and shape is noticeable. By increasing the cooling rate, the formation of larger particles is observed, however, the formation of particles smaller than 10 µm is also distinguishable. Furthermore, the large particles are not completely coalesced to spheres, as the cooling rate, again, limits the growth mechanism by accelerating the crystallization process.

Considering an increase in the stirring speed (see Figure 5c,d), the lower cooling rate shows a large variety in the resulting particle shapes, similar to Figure 4c. However, the larger particles are more likely to show a spherical structure, whereas grape-shaped structures are observed for particles with sizes below 30 µm. The increase in the cooling rate yields the formation of potato-shaped particles regardless of the particle size. Similar to Figure 4d, the large particles are to some extent covered with extremely small ones.

**Figure 6:**
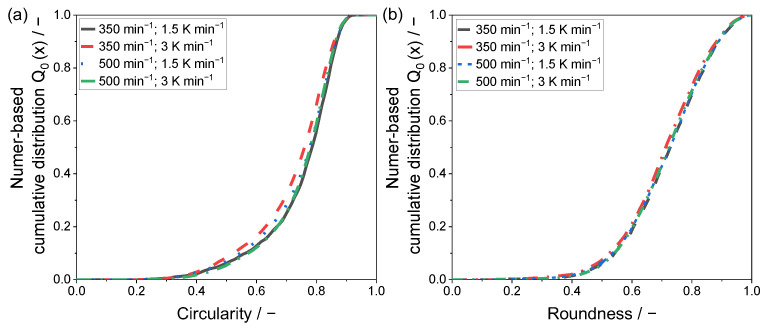
Number-based shape factor distributions of the precipitated PEI samples with a polymer concentration of 10 wt.% for (**a**) circularity and (**b**) roundness.

**Figure 7:**
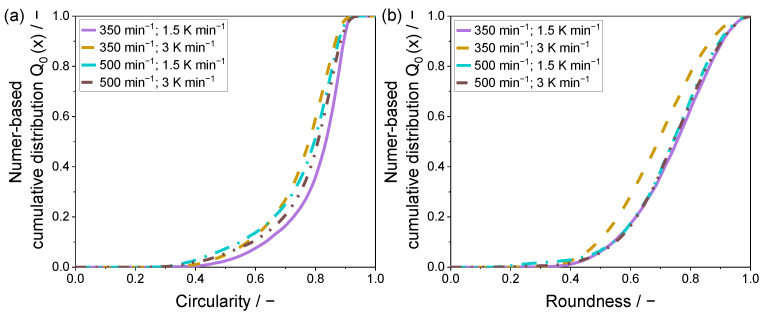
Number-based shape factor distributions of the precipitated PEI samples with a polymer concentration of 15 wt.% for (**a**) circularity and (**b**) roundness.

For a quantitative evaluation of the resulting particle shapes, a two-dimensional analysis by means of light microscopy was applied. The determined values for the shape factors circularity and roundness are given within a number-based cumulative distribution in Figure 6 and Figure 7.

The lowest sphericity is observable for the powder sample precipitated at 350 min^−1^ and a cooling rate of 3 K min^−1^, confirming the analysis of the SEM images. Regarding the circularity of the precipitated powder samples, a modest difference can be determined in between the remaining samples. Yet, the mean circularity c_50,0_ shows values of 0.77 (500 min^−1^) and 0.78, whereas the c_90,0_ shows a superior conformity of 0.86. This observation can be validated by the recorded SEM images, where the differences in between the samples displayed in Figure 4a,c,d are modest.

Considering particle roundness, the variation of the applied process parameters showed no significant impact on the resulting shape factor.

Figure 7 displays the number-based cumulative shape factor distributions of the 15 wt.% powder samples, precipitated within the stirred autoclave.

Strikingly, independent of the used polymer concentration, the similar distributions are determined for both shape factors, circularity and roundness. However, considering the circularity, the differences are larger compared to Figure 6a.

As visible in Figure 7a, the increased stirring speed results in the formation of particles with a mean circularity c_50,0_ of 0.79 and 0.81, depending on the cooling rate. Thus, lower cooling rates slightly decrease the circularity, as the precipitation is retarded, which, in combination with the major shear forces induced, forms a higher amount of non-spherically shaped fine fractioned particles. This observation is confirmed by the SEM images in Figure 5c,d.

For the lower stirring speed, the opposite trend can be determined, as the lower cooling rate yields superior circularity distributions, underlining the monomodal volumetric particle size distribution displayed in Figure 3 and Figure 5a. The mean circularity c_50,0_ values for the two samples precipitated with the decreased stirring speed, were 0.77 and 0.83, which shows a strong variation.

Considering the roundness shape factor, the parameter combination of 350 min^−1^ 3 K min^−1^ showed by far the lowest performance, as seen in Figure 7b, with a mean roundness r_50,0_ of 0.69. The remaining powder samples did not show significant differences, whereas the samples produced with a stirring speed of 500 min^−1^ were nearly identical, having a mean roundness r_50,0_ of 0.74. A marginally superior roundness distribution can be seen for the parameter combination of 350 min^−1^ and 1.5 K min^−1^, as the mean roundness r_50,0_ was 0.75.

In general, the higher the polymer fraction, the higher the impact of the process parameters on the resulting particle shape, as the particles formed are larger. Furthermore, the higher the applied stirring speed, i.e., the induced shear forces during the droplet formation process, the lower the impact of the cooling rate, as droplet breakup dominates. The SEM images presented by Wang et al. [19] reveal irregularly shaped PEEK microparticles with a rough surface structure, confirming the superior morphology of the precipitated PEI microparticles presented in this contribution.

At lower stirring speeds, the droplet formation process is predominantly controlled by the cooling rate, leading to enhanced shape factor distributions.

### 3.4. Structural Anaylsis

**Figure 8:**
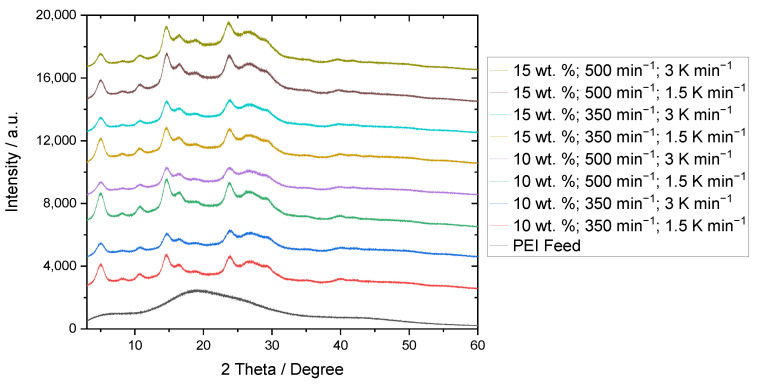
X-ray diffractograms of the precipitated PEI powder samples and PEI feed material displayed from 3 degrees to 60 degrees 2 Theta.

One major benefit of LLPS and precipitation is the possibility of the manipulation of thermal and structural product properties. This is especially true for the initially amorphous polyetherimide feed material (see black curve in Figure 8).

The structural analysis of the precipitated PEI powder samples has already been addressed in [21]. There, the presence of polymorphism was found. Nelson et al. [33] and Hsieh et al. [34] similarly investigated the crystalline structure of the modified polyetherimide material, however, they used the solvents n-methyl pyrrolidone and methylene chloride. Depending on the solvent and procedure, differing reflexes were observed in X-ray diffraction. According to Figure 8, the position of the reflexes obtained for the solvent treatment with acetophenone is consistent, independent of the applied process parameters.

### 3.5. Thermal Anaylsis

**Figure 9:**
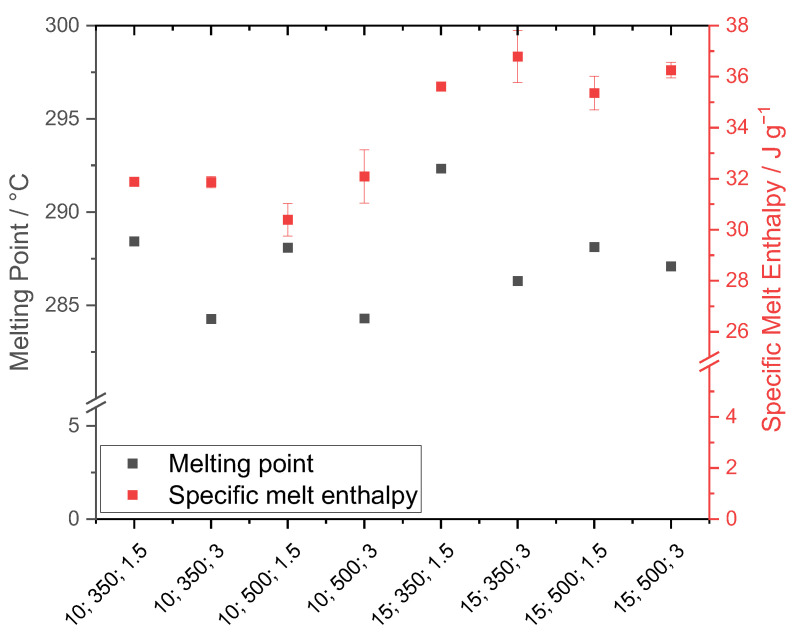
Analysis of the melting temperature and specific enthalpy of fusion of the precipitated powder samples (Labelling: polymer fraction in wt.%; stirring speed in min^−1^; cooling rate in K min^−1^).

Thermal analysis was performed with respect to the melting temperature and the specific enthalpy of fusion (Figure 9). The x-axis shows the used process parameters, although the units are not included for spatial reasons.

For the powder samples precipitated in the stirred autoclave, a noticeable dependence of the melting temperature from the cooling rate is observable. As the cooling rate decreases, the melting temperature significantly increases throughout the respective parameter combinations. Hence, lower cooling rates induce higher thermal properties, as the amount of time spent in the metastable region is increased and thus, the formation of larger crystalline structures is triggered.

Furthermore, the melting temperature increased with an increasing polymer concentration. Similar observations were made in [16]. The stirring speed instead had a reduced impact on the thermal properties, as the melting temperature showed no significant variation at increased stirring speed. For the 10 wt.% samples, the impact of stirring was minor, as no difference could be observed. However, for a polymer concentration of 15 wt.%, under an applied cooling rate of 1.5 K min^−1^, the stirring speed showed a significant impact. The authors assume that the high amount of fine fractioned particles in the concerned powder samples reduced the melting point temperature, as less energy is required for the melting process compared to larger particles.

Regarding the specific enthalpy of fusion, an almost linear trend can be observed. For the 10 wt.% powder samples, the specific melting enthalpy is constant at around 31 J g^−1^, with standard deviations of ±0.45 J g^−1^. For the 15 wt.% polymer fraction, all samples show enthalpies from 35 to 36 J g^−1^, whereas the standard deviation of the powder sample 15 wt.% 350 min^−1^ 3 K min^−1^ israther high (±1.02 J g^−1^).

### 3.6. Flowability Anaylsis

**Figure 10:**
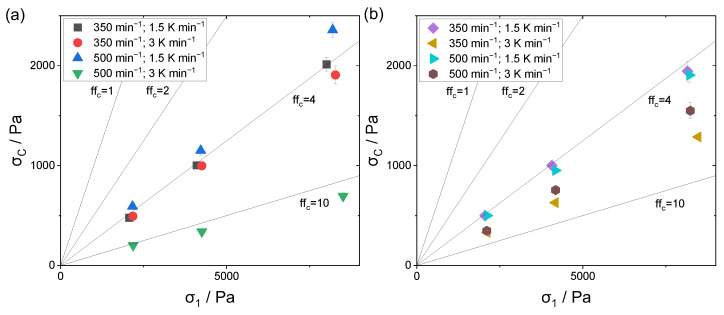
Measurement of the flowability behavior for (**a**) 10 wt.% polymer concentration and (**b**) 15 wt.% polymer concentration.

The investigation of the flowability was performed using a ring shear tester, where the resulting $ff_{c}$ factor describes the flowability class according to Jenike. Figure 10a,b displays the respective measured unconfined yield strengths $\sigma_{c}$ and consolidation stresses $\sigma_{1}$. The solid black lines mark the characteristic $ff_{c}$ factors. According to Jenike, flow factors of 4 and above characterize a properly flowing powder material, whereas values below 4 show cohesive behavior. For flow factors above 10, the material is free flowing.

Considering the analysis of the 10 wt.% polymer concentration in Figure 10a, the lowest flow factor observed is 3.5. This was caused by the broad particle size distribution of 1.4 in combination with the high amount of fine fraction, as the latter has a non-spherical morphology, limiting the flow behavior. The monomodally distributed powder sample had a proper flow factor of 4.0, whereas the higher cooling rate at the constant stirring speed showed a marginally increased flowability of 4.4. With respect to the displayed standard deviations, no distinct difference was observable between the samples depicted with black and red symbols. One can assume that the marginally broader particle size distribution along with a modest amount of fine fractioned particles slightly enhances the flowability behavior, as a densely packed powder bed is achievable. Considering the shape analysis, the positive impact of a spherical particle shape compared to a potato shape was not distinguishable, and the particle shape did not have a major impact on the flow behavior.

A superior flowability behavior with a flow factor of 12.3 is noticeable for the sample outlined with green symbols. Here, both the rotation speed and the cooling rate were increased, resulting in the highest amount of median fractioned particles having a total size range of 2 to 105 µm. As the shape analysis did not reveal a superior shape, the impact of the latter has to be considered with respect to the particle size analysis.

For the powder samples with a polymer concentration of 15 wt.%, all flow factors are located within the region of 4 to 10. Similar to the 10 wt.% samples, the monomodally distributed sample had one of the lowest flow factors. Considering the variation in the stirring speed at a constant cooling rate, the resulting observable flowability remained the same. By increasing the cooling rate at an elevated stirring speed, the flowability was improved, due to a reduced fine fraction.

However, the powder sample with the smallest fine fraction and largest amount of median fractioned particles, as well as a narrow particle size distribution (size range: 1 to 120 µm; span: 1.2) (golden symbols), again showed a superior flowability behavior, although the shape analysis revealed it to have by far the lowest number-based form factor. As already assumed above, for the analysis of the flowability behavior, both the particle size and the particle shape distribution have to be considered.

To summarize, the larger the fine fraction in relation to the median fraction, the worse the resulting flowability. Moreover, a span below 1, indicating a monomodal distribution, leads to a decreased flowability behavior, as due to the uniformity of the particle sizes, only a modest packing density is achievable.

## 4. Multivariate Correlation

**Figure 11:**
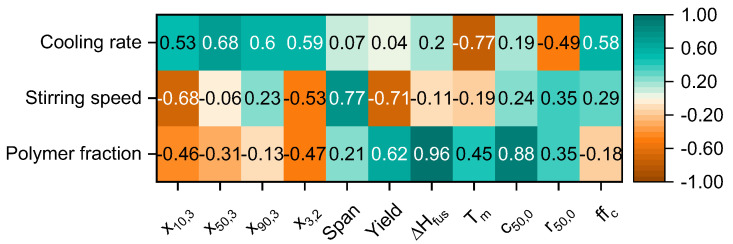
Multivariate correlation matrix of the three main components and the resulting characteristic particle properties.

The multivariate correlation matrix containing the Pearson’s Linear Correlation Coefficient *ρ*(*a*,*b*) is presented in Figure 11. Positive correlations are marked in green, negative ones are depicted in orange. Color intensity signals the magnitude of the correlation. The correlations of the three main components (e.g., polymer fraction, stirring speed and cooling rate) are analyzed first, and afterwards the reciprocal correlation of the particle properties is investigated.

The strongest correlations with the polymer fraction are found with the specific melting enthalpy (0.96) and the mean circularity c_50,0_ (0.88). An increase in the polymer fraction significantly leads to a uniform increase in the specific melting enthalpy, as larger particles are formed consisting of more dense crystalline structures. Furthermore, with an increasing polymer fraction, the mean circularity of the polymer particles increases with a correlation factor of 0.88. The amount of small particles that are aggregated with larger ones is decreased, as the volumetric distribution of small to larger particles is less bimodal. Moreover, larger particles are formed with respect to the fine and mean fraction, which is confirmed by a modest correlation for x_10,3_ = −0.46 and a weak correlation for x_50,3_ = −0.31.

Considering the stirring speed, the most pronounced correlations are found for the span (0.77) and the fine fraction x_10,3_ (−0.68). With an increasing fine fraction, an increase in the span is observed. The pronounced impact of the stirring speed on the particle sizes could be related to the enhanced stresses that are transferred into the liquid–liquid dispersed system leading to an increase in the droplet breakup rate, thus yielding smaller particle sizes.

As both the polymer faction and the stirring speed are strongly correlated with the resulting particle morphology, the cooling rate additionally affects the melting temperature. The latter has a strong negative correlation of −0.77. Thus, the higher the cooling rate, the lower the melting temperature. The accelerated cooling process limits the growth of the individual crystals resulting in reduced melting temperatures. This hypothesis is confirmed by the X-ray diffractograms, where the reflexes for powders produced with a cooling rate of 1.5 K min^−1^ are characterized by a smaller full width at half maximum as compared to the samples obtained with a cooling rate of 3 K min^−1^. Furthermore, the cooling rate was found to be negatively correlated with the mean roundness r_50,0_ (−0.49), which might be explained by the limited development of completely coalesced structures due to rapid cooling.

The flowability is positively correlated with the cooling rate (0.58), as an enhanced cooling rate yields the formation of larger particle sizes. Consequently, the flowability is improved, as small particles, which would limit the flowability, are less abundant.

**Figure 12:**
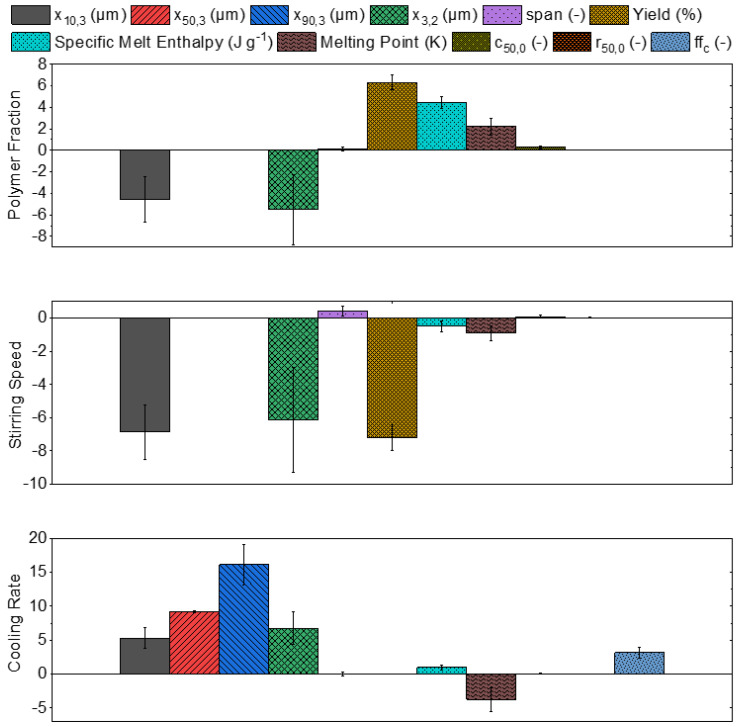
Quantitative analysis of the resulting characteristic particle properties.

By analyzing Figure 11, the (qualitative) correlation strength and direction between the process parameters and the resulting particle properties was determined. In order to quantify the amount of particulate property change based on the increase in the respective process parameter, Figure 12 was used. The units of the y-axis (polymer fraction, stirring speed, and cooling rate) correspond to the unit of the respective particle property, so there is no universal unit displayed. The change in the property can be analyzed with respect to its absolute value. If no value is assigned for a particle property, it means that no explicit influence was recognizable.

The stirring speed had the strongest impact on the fine fraction particle size (−6.9 µm, to a minimum x_10,3_ of 12.2 µm), followed by the weaker impact of the polymer fraction (−4.6 µm). This simultaneously affects the Sauter diameter x_3,2_ (−5.5 µm, −6.1 µm, and +6.7 µm), where the percentage decrease is smaller as the Sauter diameter initially has larger particle sizes. The cooling rate (+5.3 µm) has a positive impact on the fine fraction particle size. Despite this, the fine fraction size is predominantly controlled by the polymer fraction and stirring speed.

The mean (+9.2 µm) and coarse (+16.1 µm) fraction are exclusively affected by the cooling rate, as both are increased at an accelerated cooling rate.

No significant impact on the span can be determined. Yet, an increase in the stirring speed leads to an increase in the span (+0.40). As the span is already in an advantageous range, regardless of the applied process parameters, this effect is not that important.

For the yield, opposite trends can be observed, as an increase in the polymer fraction enhances the yield (+6.3%), whereas an increase in the stirring speed decreases the yield (−7.2%). As a result, the yield of each of the two samples is nearly identical.

The specific melting enthalpy is predominantly dependent on the polymer fraction (+4.5 J g^−1^), whereas the impact of both the stirring speed (−0.5 J g^−1^) and cooling rate (+0.9 J g^−1^) is small. Considering the melting point temperature, the strongest impact can be observed on the cooling rate (−3.8 °C), whereas the polymer fraction (+2.2 °C) shows a modest impact. Even though the process parameters reveal opposite impacts, they do not affect each other. The influence of stirring speed (−0.9 °C) is negligible.

For both form factors, circularity and roundness, no significant impact of the process parameters can be found. Even though, according to Figure 11, the polymer fraction has a correlation coefficient of 0.88, the absolute value for the increase in the mean circularity is +0.26, showing the relevance of a supplementary analysis of the absolute values.

The flowability is solely dependent on the applied cooling rate (+3.2), as an accelerated cooling rate enhanced the flowability of the powder samples. The impact of the polymer fraction and stirring speed was not clearly determinable, thus, no values were assigned.

## 5. Conclusions

With this study, LLPS and precipitation have been demonstrated to be a viable novel approach for the production of semi-crystalline polyetherimide microparticles, a promising high-temperature thermoplast feedstock for PBF-AM. Moreover, a tailored control of the resulting particle properties by the adjustment of the applied process parameters, e.g., stirrer rotation speed, cooling rate, and polymer concentration, is possible. The above-mentioned parameters are individually controllable to achieve the most proficient particle properties.

The largest impact on the enhancement of the thermal properties was observed for the cooling rate, which determines the size of the crystal structures formed. Thus, larger crystal structures lead to an advantageously increased melting temperature.

The size distribution of the precipitated particles is mainly controlled by the stirring speed, which determines the amount of shear force induced during the droplet formation process. Higher stirring speeds lead to a moderate decrease in particle size. The largest impact of the stirring speed can be observed on the formation of the fine and median fraction, as the amount of fine fractioned particles is especially increased.

The particle morphology is affected by the combination of parameters applied, as two-way interactions can lead to both decreased and increased frequencies of particle morphology. The combination of low cooling rates and stirring speeds is favorable, whereas high cooling rates at low stirring speeds is adverse. Contrary observations can be made for higher stirring speeds, where the differences in the particle morphology are smaller.

In this paper, the authors propose a guideline for the tailored control of particle properties based on a two-level factorial design of experiments. This study focused on enhancing the material variety of high-performance polymers available for PBF-LB/P. Foremost, a semi-crystalline structure is desirable, as this improves the melting behavior of the polymer. Upon melting of the polyetherimide particles, the initially amorphous structure of the thermoplast is regained and there is a lower shrinkage upon solidification and formation of the amorphous part compared to thermoplasts such as PA12 that yield semi-crystalline parts. The extrinsic properties of the produced semi-crystalline polyetherimide particles are beneficial for powder spreading and processing and the amorphous solidification of the part is beneficial for its dimensional accuracy and mechanical properties.

## Figures and Tables

**Table 1:** Factor plan for the stirred autoclave.

A: Polymer Fraction/wt.%	B: Stirring Speed/min^−1^	C: Cooling Rate/K min^−1^
10 (+)	350 (+)	1.5 (+)
15 (−)	500 (−)	3 (−)

**Table 2:** Two-level factorial design of experiments for the stirred autoclave.

No.	A	B	C
1	+	+	+
2	+	+	−
3	+	−	+
4	+	−	−
5	−	+	+
6	−	+	−
7	−	−	+
8	−	−	−

**Table 3:** Yield resulting from the performed experiments.

No.	Input Mass/g	Output Mass/g	Yield/%
1	200	187.1	93.6
2	200	184.9	92.5
3	200	170.6	85.3
4	200	181.1	90.5
5	300	301.9	100.62
6	300	298.7	99.58
7	300	277.3	92.4
8	300	275.9	91.9

## Data Availability

The data that support the findings of this study are available from the corresponding author, upon reasonable request.
